# Neuroimaging Studies of Chronic Prostatitis/Chronic Pelvic Pain Syndrome

**DOI:** 10.1155/2022/9448620

**Published:** 2022-05-04

**Authors:** Yifan Zhao, Jiaqi Lin, Ye Dong, Zilei Tian, Yan Ye, Ziyang Ma, Shengli Xia, Xiaopeng Huang, Diang Chen, Peihai Zhang

**Affiliations:** ^1^Chengdu University of Traditional Chinese Medicine, Chengdu 610072, China; ^2^TCM Regulating Metabolic Diseases Key Laboratory of Sichuan Province, Hospital of Chengdu University of Traditional Chinese Medicine, Chengdu 610072, China

## Abstract

Evidence shows that chronic prostatitis/chronic pelvic pain syndrome hugely impacts the body and mind. The central mechanisms in patients with CP/CPPS resulted in increased attention as neuroimaging techniques developed. This review investigated the study design and major neuroimaging findings in CP/CPPS patients to provide comprehensive evidence. Seven databases were searched and screened: PubMed, EMBASE/SCOPUS, Cochrane Library Database, China National Knowledge Infrastructure, VIP, Wanfang, and China Biology Medicine disc. Nine studies were eventually included in the analysis. The results demonstrate that the insula, anterior cingulate gyrus, postcentral gyrus, and precuneus are significantly associated with CP/CPPS patients' pain feelings and cause dysregulation of painful emotions, lowering patients' tolerance to stimulus.

## 1. Introduction

Chronic prostatitis/chronic pelvic pain syndrome (CP/CPPS) is a common urological disorder characterized by pelvic pain or discomfort and abnormal urination [1]. Epidemiological studies showed that the global prevalence of CP/CPPS ranges between 9% and 16% [2]. Meanwhile, approximately 60% of CP/CPPS patients suffer from severe depression [3]. More importantly, long-term chronic pain leads to a lower quality of life to levels comparable to cardiovascular diseases, including severe congestive heart failure [4, 5]. The National Institutes of Health (NIH) classified prostatitis into acute bacterial prostatitis (type I), chronic bacterial prostatitis (type II), chronic prostatitis/chronic pelvic pain syndrome (type III), and asymptomatic prostatitis (type IV) based on different causes of prostatitis [1]. Types I and II have a bacterial infection, and type IV has evidence of inflammation. CP/CPPS is the most common type of chronic prostatitis [1], and it lacks objective and specific biological indicators when compared to the other three types.

The advancement of neuroimaging techniques has aided in identifying functional/structural brain alterations in patients with chronic pain disorders, including CP/CPPS and chronic low back pain [6]. The CP/CPPS mechanisms studies have gradually shifted to investigating critical pathological features. A functional magnetic resonance imaging (fMRI) study [7], for example, demonstrated that anterior insula activity increased in CP/CPPS patients compared to healthy individuals and was correlated with clinical pain intensity. Meanwhile, in CP/CPPS patients, the right anterior insula and parietal regions were more specifically active during spontaneous pain. Using structural magnetic resonance imaging (MRI) [8], researchers discovered that CP/CPPS patients had lower gray matter volume in the anterior cingulate cortex in the dominant hemisphere. These neuroimaging studies demonstrate that CP/CPPS is not only a genitourinary disorder but also has abnormal changes in the brain function and structure. This scattered evidence needs further exploration to better understand the critical pathological changes in CP/CPPS. Therefore, this study aimed to review the critical brain regions involved in pain regulation in CP/CPPS and compare functional brain activity/structural differences between CP/CPPS patients and healthy controls.

## 2. Materials and Methods

### 2.1. Eligibility Criteria

Inclusion criteria are as follows: (1) controlled trial; (2) patients with CP/CPPS (type III); (3) pain in the perineum and pelvic area; (4) disease course of at least two weeks; (5) outcome measures including pain in the perineum and pelvic area and fMRI/MRI data.

Exclusion criteria are as follows: (1) Animal experiments, case reports, pharmacological-pharmacokinetic studies, or literature reviews; (2) study subjects had urological disorders other than CP/CPPS, including erectile dysfunction, infertility, and other male diseases; (3) history of using alcohol or any drug that influences the brain function or structure such as psychotropic drugs.

### 2.2. Information Sources

Computer searches of seven databases (PubMed, EMBASE/SCOPUS, Cochrane Library Database, China National Knowledge Infrastructure, Vipshop, Wanfang, and China Biology Medicine Disc) were conducted for articles published since the database's inception until September 1, 2021, for CP/CPPS neuroimaging studies based on MRI/fMRI technology.

### 2.3. Search Strategy

The search was performed using a combination of subject headings and free words. The English search terms include the following: (“prostatitis” [MeSH Terms] OR “prostatitis” [All Fields] OR (“chronic” [All Fields] AND “prostatitis” [All Fields] AND “chronic”[All Fields] AND “pelvic” [All Fields] AND “pain” [All Fields] AND “syndrome” [All Fields]) OR “chronic prostatitis chronic pelvic pain syndrome” [All Fields] OR(“chronic nonbacterial prostatitis” [All Fields] AND “prostatodynia” [All Fields])) AND (“magnetic resonance imaging” [MeSH Terms] OR (“magnetic” [All Fields] AND “resonance” [All Fields] AND “imaging” [All Fields]) OR “magnetic resonance imaging” [All Fields] OR “MRI” [All Fields]) AND (“brain” [MeSH Terms] OR “brain” [All Fields]). Supplementary Materials include the corresponding search terms used in the Chinese search. Chinese search terms are listed in Supplementary Table 1 in Supplementary Materials.

### 2.4. Selection Process

Two fully independent evaluators screened the literature based on the inclusion and exclusion criteria, respectively. First, duplicates and those who did not meet the inclusion criteria were eliminated by reading the title and abstract; second, the full text was read again, and the results were cross-checked for potential inclusion. Any disagreement was solved by the two evaluators through discussion and negotiation, and if no agreement was reached, a third evaluator was asked to review the literature further. If the authors have not provided the activation zone coordinates in the text, the evaluator should send emails to the authors to ask about data; if the authors do not respond within two weeks, the study will be excluded from the ALE analysis.

### 2.5. Data Collection Process

**Figure 1:**
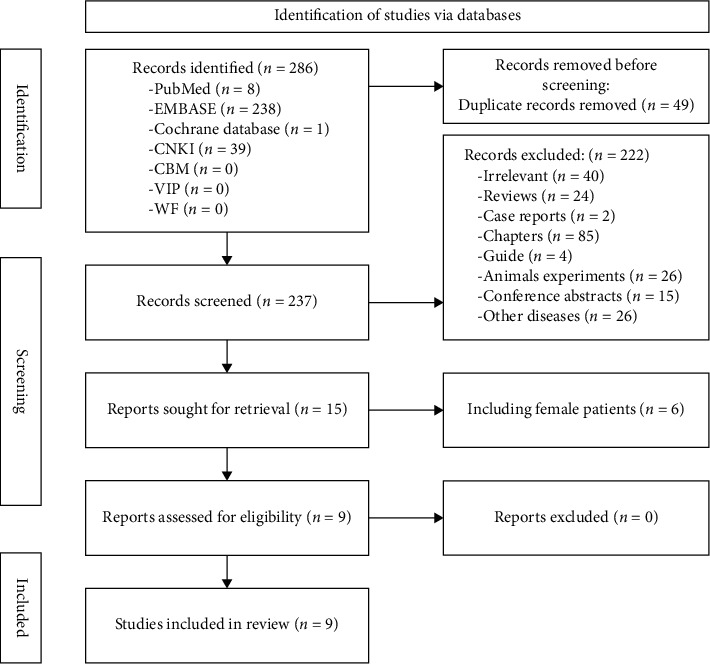
Flowchart of literature screening.

The following data were extracted from the literature based on the inclusion criteria: (1) general information: title, author, journal, country or region of origin, and publication date; (2) study characteristics: study subject sample size, age, handedness, education, duration of illness, type of trial, CP/CPPS diagnostic criteria, baseline and intervention criteria, imaging methods, clinical/behavioral indicators, and emotional indicators; (3) nodal indicators: abnormal brain areas and trends in CP/CPPS, coordinates, and clinical correlation. The current systematic review was performed following the PRISMA checklist.  Figure 1 depicts the flowchart of literature screening.

### 2.6. Data Analysis

The likelihood estimation (ALE) is a coordinate-based functional analysis approach proposed by Turkehaub et al. [9] to localize brain areas by performing three-dimensional Gaussian function smoothing and alignment tests on the relevant coordinates incorporated into the literature. Eickhoff et al. [10] improved the original statistical model by changing the fixed model into a random model, making the analysis results more objective and accurate.

Specifically, (1) the Talairach coordinates were converted to MNI (Montreal Neurological Institute) coordinates using the coordinate conversion function of the Icbm2tal software. The coordinates were grouped and entered into the Ginger-ALE software according to the data entry method in the ALE manual. (2) Data were analyzed, and ALE maps were calculated using Ginger-ALE 2.3 software. Based on the peak maximum activation coordinates, a three-dimensional Gaussian distribution was modeled with the following parameters: the Gaussian filtered full width half maximum (FWHM) value was used as the default setting based on the number of subjects, and the software calculated the whole-brain ALE distribution. Cluster thresholds were *p*-FWE <0.05, voxel thresholds were *p*-uncorrected < 0.05, and the number of thresholding permutations was 5000. Brain region image results were presented using the DPABI toolbox (https://rfmri.org/).

## 3. Results

### 3.1. Study Information

A total of 247 English and 39 Chinese records were obtained through preliminary retrieval. After checking titles and abstracts, 277 duplicates and those that did not meet the inclusion criteria were eliminated; 0 were excluded by further reading the full text. Finally, nine studies were included (S1–S9); those published between 2011 and 2021 were included in this review (Table 1; Table 2). Five of these studies were conducted in China (S4; S6; S7; S8; S9), two in the United States (S1; S3), and 2 in Switzerland (S2; S5). No risk of bias was found in the included references.

### 3.2. Participants

The nine studies enrolled 431 study subjects, including 233 patients with CP/CPPS and 198 healthy subjects (HC). Eight of the nine studies (S1–S4; S6–S9) compared CP/CPPS patients with HC, while 1 study (S5) recruited only CP/CPPS patients. The maximum sample size for a single group of patients with CP/CPPS 31 was 19 in nine studies.

### 3.3. Control Group

One of the nine studies (S5) used acoustic magnetic therapy in the treatment group and sham stimulation in the control group. The other eight studies used healthy subjects as control groups.

### 3.4. Intervention Details

All studies required patients to be right-handed; two studies (S3; S7) required subjects to empty their bladders before the MRI examination; two studies (S4; S6) required subjects to have a visual analog score of 0 before the MRI examination; six studies (S1; S3–S6; S7) required subjects to be resting with their eyes closed but not asleep; three of these studies (S2; S8; S9) did not specify any patient requirements.

### 3.5. Magnetic Resonance Imaging Techniques and Analysis Methods

All studies adopted magnetic resonance imaging (MRI) to detect changes in the structural/functional activity of the brain of CP/CPPS patients. Five of the nine studies examined structural changes. Two studies (S8; S9) used diffusion tensor imaging (DTI) techniques to compare the global functional connectivity of brain white matter structures in CP/CPPS patients and healthy subjects. Two studies (S1, S7) compared regional gray matter (location of neuronal vesicles) and white matter (location of axonal tracts) features and functional brain connectivity in patients with CP/CPPS to those in healthy subjects using DTI and high-resolution T1-weighted MRI. Another study (S3) compared the functional connectivity of motor cortex areas directly controlling the pelvic floor in patients with CP/CPPS and healthy subjects using T1- and T2-weighted MRI image analysis. Two studies (S4; S6) used rs-fMRI to assess the mean regional homogeneity (ReHo) values of voxels in standardized brain regions. One study (S5) investigated the possibility of longitudinal cerebral blood flow (CBF) changes in CP/CPPS patients using arterial spin labeling (ASL) techniques. Another study (S2) investigated the role of the anterior cingulate cortex in chronic pelvic pain syndrome using T1-weighted MRI.

### 3.6. Brain Alterations in Patients

The findings of nine studies revealed that patients with CP/CPPS had changes in 39 brain regions, primarily the left anterior cingulate cortex, right anterior cingulate cortex, right insula, left postcentral gyrus, left precentral gyrus, and left precuneus.

Four studies (S2, S4, S5, S6) discovered changes in the anterior cingulate cortex (ACC) in CP/CPPS patients versus healthy subjects. Two of these studies (S4, S6) revealed changes in the ACC function, with a significant decrease in the ReHo value of the bilateral ACC and a significant negative correlation between the degree of activation of the bilateral ACC and the National Institutes of Health-chronic prostatitis symptom index (NIH-CPSI) scale pain score. Another study (S2) demonstrated changes in ACC structure, including a significant decrease in volume and an increase in density in the gray matter of the left ACC brain. Another study (S5) revealed that CBF was downregulated in the anterior cingulate cortex of patients.

Three studies (S1, S3, S4) found insula (INS) changes in CP/CPPS patients versus healthy subjects. One study (S4) revealed structural changes in the INS and a significant reduction in ReHo in the insula cortex of CP/CPPS patients. Two studies (S1, S3) correlated the functional changes in INS, functional activation within the patient's right anterior insula, and pelvic-motor connectivity to the right posterior insula with pain intensity in CP/CPPS.

Three studies (S1, S8, S9) found changes in the structure of the postcentral gyrus (PoCG), precentral gyrus (PreCG), and precuneus (PCUN) in CP/CPPS patients, all with significant increases in the values of the local efficiency attributes. The overall efficiency value of the left PoCG increased. The NIH-CPSI scale total score, the pain and discomfort symptom score, and the impact of symptoms on quality of life score all had a positive correlation with the local efficiency of the left PCUN.

Two studies (S1, S5) found increased CBF in the left dorsomedial and right dorsolateral prefrontal cortex resulting in functional changes in the dorsolateral prefrontal cortex (DLPFC).

Two studies (S4, S6) revealed changes in thalamus (THA) function, including significantly higher ReHo values and homogeneity in the right thalamic area and a significant positive correlation between the degree of activation in the right thalamic area and the NIH-CPSI scale pain score.

Two studies (S5, S7) found changes in medial prefrontal cortex (mPFC) function and increased CBF in patients' left medial and right dorsolateral prefrontal cortex.

Two studies (S7, S9) revealed structural changes in the middle frontal gyrus, orbital (ORBmid), median, and paracingulate gyrus (DCG), supplementary motor area (SMA), right superior temporal gyrus (STG), left superior marginal gyrus (SMG), left superior parietal gyrus (SPG), left posterior cingulate gyrus (PCG), and paracentral lobule (PCL) in CP/CPPS patients. The right middle frontal gyrus (orbit) is inefficient in CP/CPPS patients, implying a disruption in the connection of the entire brain to the right middle frontal gyrus (orbit). Local efficiency attribute values increase significantly in the left paracingulate gyri, supplementary motor area, right superior temporal gyrus, left supramarginal gyrus, left superior parietal gyrus, and left posterior cingulate gyrus. The overall efficiency of the left paracingulate gyrus and left posterior cingulate gyrus is improved. The NIH-CPSI scale total score, pain and discomfort symptom scores, and symptom impact on quality of life scores were all positively correlated with the right supplementary motor area (Table 3).

**Figure 2:**
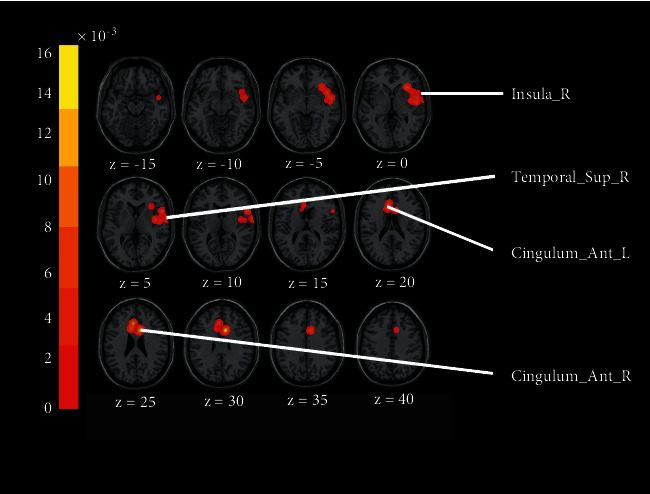
ALE analysis results.

A total of 207 subjects were included in the ALE analysis, consisting of 9 studies, 29 foci, and a total of 2 significant clusters. Cluster 1 included the insula and superior temporal gyrus, and cluster 2 consisted mainly of the cingulate gyrus region (Table 4 and Figure 2).

## 4. Discussion

### 4.1. A Narrative Review of Nine Studies on MRI of the Brain in Patients with CP/CPPS

A narrative review of nine studies on brain MRI in CP/CPPS patients revealed a close association of clinical pelvic pain symptoms with functional or structural changes in brain regions, including left ACC, right ACC, right INS, right STG, and left PCUN in male CP/CPPS patients.

This systematic review found structural changes in the ACC in CP/CPPS patients in four studies (S2, S4, S5, S6). The ACC is a critical brain region component of the neural substrate involved in pain processing [11]. Lin et al. (S4) found that in CP/CPPS patients, regional homogeneity (ReHo) was significantly decreased in the ACC, insula cortex, and right medial prefrontal cortex but significantly increased ReHo in the brainstem and right thalamus. These findings demonstrate that nociceptive information is correlated to these brain regions. Furthermore, emerging evidence shows that noxious stimuli are transmitted indirectly to the ACC via at least three major projection systems [12]. The first comes from the thalamus, where nociceptive input from the medial thalamus is received by ACC neurons [13, 14]. The amygdala sends the second nociceptive message to the ACC. Other pain-related cortical areas, including the insula cortex, are the third source of nociceptive input to the ACC. Lin et al. (S4) showed decreased ReHo in bilateral ACCs and INCs, indicating impaired downstream pain inhibition in CP/CPPS. Furthermore, an impaired pain modulation system could explain the pain symptoms of CP/CPPS by decreasing downstream pain inhibition or increasing pain vulnerability. It is also possible that AC1-triggered cAMP signaling contributes to synaptic changes associated with chronic pain in the ACC. Mordasini et al. (S2) demonstrated a significant decrease in brain gray matter volume and an increase in density in CP/CPPS patients with left ACC, suggesting that this mechanism is involved.

Three studies (S1, S3, S4) found structural changes in the INS of CP/CPPS patients. Two studies (S1, S3) found functional activation of the right anterior insula in CP/CPPS patients. Mounting evidence from pain studies shows that the insula capitol cortex, which plays a critical role in chronic pain, is the brain region most consistently activated [15–17]. The insula cortex plays a role in both the sensory-discriminative and affective-motivational aspects of nociceptive processing [18]. According to the findings of Xu et al. (S9), the anterior insula (AI) cortical area was primarily associated with pain-induced affective feelings, whereas the posterior insula (PI) cortical area was associated with sensory information of nociception [19]. Pain information elicits an emotional response through this pathway, which activates the downstream pain modulation system. Farmer et al. (S1) demonstrated that pain information in the insula cortical area might influence brain activity other than the primary clinical symptoms of CP/CPPS. The posterior insula cortical area receives nociceptive afferents primarily from the ventral posterior inferior nucleus and the thalamus ventromedial posterior nucleus, both of which receive input from spinothalamic neurons of lamine I [20]. The degree of activation of the posterior insula cortical area was most consistent with chronic pain progression [21]. Kutch et al. (S3) revealed that the functional connectivity between the motor cortex and the posterior insula is potentially one of the most critical markers of the altered brain function in CP/CPPS patients, representing changes in the integration of visceral sensory and motor processing.

Three studies (S1, S8, S9) found structural changes in PCUN in CP/CPPS patients. Positive correlations were found between the local efficiency of the left PCUN and the NIH-CPSI scale total score, pain and discomfort symptom score, and symptom impact on quality of life score. These findings demonstrate that the higher the local efficiency of the left PCUN, the more severe the patient's pain symptoms. The PCUN is the functional core of the posterior DMN [22], and it is in charge of self-processing and internal sensing, as well as pain sensitivity and pain threshold determination.

### 4.2. Discussion of the Results Based on ALE

The ALE result suggests significant INS, STG, and ACC changes in patients with CP/CPPS. In S9, the ratio of STG network efficiency is more significant in patients than in healthy individuals. Robust evidence [23] suggests that the STG may play a role in encoding experimental pain memory and that the STG is involved in memory bias associated with experimental pain memory. Stimulation of the STG affected the motivational-emotional component of the pain experience but not the sensory discrimination component, highlighting the role of the cortical region in the emotional memory of experimental pain.

Chronic pelvic pain syndrome is a centralized pain syndrome associated with central nervous system changes. Abnormal regulation and output of the hypothalamic-pituitary-adrenal (HPA) axis are frequently related to centralized pain disorders. The HPA axis is the primary stress response system, and its activation induces cortisol production and immune response dampening. Patients with centralized pain syndromes frequently have hypercortisolism or hypocortisolism, and evidence of altered downstream signaling from the HPA axis, including increased mast cell (MC) infiltration and activation, can cause sensitization of nearby nociceptive afferents. Increased peripheral input via nociceptor activation can result in “hyperalgesic priming” and “wind-up,” which may eventually cause central sensitization via long-term potentiation in the central nervous system [24]. CP/CPPS pain is not only felt near the prostatic organ but also in the L5-S2 sacral innervation area associated with vesicourethral innervation, and central sensitization of the L5-S2 sacral nerve segments may be a cause of CP/CPPS pain [25].

Studies reviewed herein revealed that critical regions played a role in pain relief. Opioid signaling in the anterior cingulate cortex, activation of midbrain dopamine neurons, and dopamine release in the nucleus accumbens are all required for the rewarding effect of pain relief [26]. The ventral anastomotic insular region (RAIC) is part of the anterior insula. A study by Zhang [27] revealed that chronic pain increases the excitability of RAIC pyramidal neurons, and activation of cannabinoid receptors within the RAIC can significantly inhibit mechanical touch-evoked pain in mice. Moreover, RAIC may be involved in regulating dopamine in the vomeronasal nucleus, thereby inhibiting injury perception [28]. Stimulation of the STG with a single pulse of TMS (virtual disruption paradigm) prevents exaggeration of memory for painful events when administering electrical pain stimulation [23].

## 5. Conclusion

The changes in brain regions of CP/CPPS patients are primarily concentrated in the left ACC, right ACC, right INS, right STG, and left PCUN. The ACC receives pain signals via the thalamus, amygdala, and insula cortex, and the regional homogeneity of bilateral ACC in CP/CPPS patients is significantly reduced. Patients with CP/CPPS have functional activation in the right anterior insula. Pain significantly impacts the anterior insula in CP/CPPS patients, with altered functional connectivity between the motor cortex and the PI. The higher the local efficiency of the left PCUN, the worse the pain symptoms in CP/CPPS patients. The network efficiency ratio was greater in STG patients than in healthy individuals, and STG was involved in the memory bias associated with experimental pain memory.

The present systematic review has some limitations. The studies included were nonrandomized controlled trials, and due to the limitations of the study design, selection and measurement bias could not be avoided. The contribution analysis of ALE suggested that cluster 1 was provided by S1, S3, and S4, of which S3 had a high contribution; Cluster 2 was provided by S2, S4, S5, and S6, of which S4 and S6 had a high contribution; S8 and S9 have not made any contribution between cluster 1 and cluster 2 (Table 1). This may be related to the quality of the references. Due to the number limitation of the included references, this study investigated the potential changes in the brain regions of CP/CPPS patients through the ALE method and provided the quality was suitable for the study, which provided ideas for future studies.

Despite the high prevalence of pain in CP/CPPS patients, current neuroimaging studies provide only a limited understanding of pain mechanisms. There is ample opportunity to advance our understanding of CP/CPPS-related pain mechanisms and future CP/CPPS treatment. More attention should be paid to these brain regions with targeted studies on targeted drugs for pain relief in CP/CPPS. The use of functional and advanced structural MRI techniques to compare differences between CP/CPPS patients and healthy individuals has the potential to advance research in this critical area.

## Figures and Tables

**Table 1:** Basic information for inclusion in the study.

No.	Author (year) country	Disease	Design	Sample capacity	Age	Intervention/control	Manipulation modality	Imaging modality	Analytical approaches	Cluster1 contribution degree	Cluster2 contribution degree
S1	Melissa A Farmer (2011) American Farmer et al., 2011	CP/CPPS	Non-RCT	19/16	36.94	—	Resting state	DTI, T1-weighted MRI, rs-fMRI	Talairach space	1	0

S2	Livio Mordasini (2012) Switzerland Mordasini et al., 2012	CP/CPPS	Non-RCT	20/20	40 ± 14	—	—	T1-weighted MRI	Talairach space	0	1

S3	Jason J kutch (2015) American Kutch et al., 2015	CP/CPPS	Non-RCT	28/27	32.7 ± 1.5	—	Resting state; participants need to empty their bladders	rs-fMRI, T2-weighted MRI, T1-weighted MRI	MNI	6	0

S4	Yusong Lin (2017) China Lin et al., 2017	CP/CPPS	Non-RCT	31/31	34.1 ± 10.3	—	Resting state, no spontaneous pelvic pain; the visual analog scale test must score 0 within 10 minutes before and after the MRI scan, respectively	rs-fMRI, T1-weighted MRI	MNI	1	2

S5	Christian Weisstanner (2017) Switzerland Weisstanner et al., 2017	CP/CPPS	RCT	30 (therapy)/30 (sham therapy)	46 ± 14	Sono-electromagnetic/sham therapy 12 weeks of treatment	Resting state	T1-weighted MRI, ASL, BOLD-MRI	MNI	0	1

S6	Yan Bai (2017) China Bai et al., 2017	CP/CPPS	Non-RCT	19/19	34.8 ± 9.6	—	Resting state; the visual analog scale test must score 0 within 10 minutes before and after the MRI scan, respectively	Rs-fMRI	MNI	0	2

S7	Shengyang Ge (2021) China Ge et al., 2021	CP/CPPS	Non-RCT	18/21	30.92 ± 7.69	—	Resting state; participants need to empty their bladders	rs-fMRI, T1-weighted MRI	MNI	0	

S8	Xinfei Huang (2021) China Huang et al., 2021	CP/CPPS	Non-RCT	19/32	38.11 ± 9.02	—	—	T1-weighted MRI, DTI	MNI	0	0

S9	Yan Xu (2021) China Xu et al., 2021	CP/CPPS	Non-RCT	19/32	38.11 ± 9.02	—	—	T1-weighted MRI, DTI	MNI	0	0

**Table 2:** Diagnostic criteria of the study.

No.	Diagnostic criteria
S1	Patients who had pelvic discomfort/pain for three or more months within the last six months; an overall score of 15 or greater of 43 points on the National Institutes of Health Chronic Prostatitis Symptom Index (NIH-CPSI), including 1 or more on the pain subscale of this index or current pain.

S2	In accordance with European Association of Urology guidelines, patients with CPPS complained about pain perceived in structures related to the pelvis for at least three months without proven infection or other obvious pathology. Inclusion criteria were a NIH-CPSI total score of 15 or greater, NIH-CPSI pain subscale eight or greater.

S3	CP/CPPS patients had to report an unpleasant sensation of pain, pressure, or discomfort perceived to be related to the bladder and/or pelvic region for most of the time during the most recent three months.

S4	Patients had complaints about pelvic pain at least three months within the last six months; the NIH-CPSI total score was 15 or greater.

S5	In accordance with the European Association of Urology guidelines, all patients with chronic pelvic pain syndrome (CPPS) enrolled in this study reported pain perceived in structures related to the pelvis for at least three months without proven infection or other related pathologies.

S6	Meets National Institutes of Health diagnostic criteria for CP/CPPS.

S7	According to the category of National Institutes of Health, CP/CPPS was defined as urological pain or discomfort in the pelvic region sustained for no less than three months during the preceding six months that is associated with lower urinary symptoms and not in consort with a urinary tract bacterial infection.

S8	Based on the NIH-CPSI, patients with at least three months of persistent pelvic pain, urinary symptoms, and/or sexual dysfunction can make the diagnosis of CP/CPPS after excluding obvious etiologies such as active UTIs.

S9	The patient had pain and discomfort in the pelvic region for more than three months; NIH–CPSI score ≥15; normal white blood cell count and negative bacterial culture on routine prostate massage.

**Table 3:** Nine studies of functional or structural brain changes in patients with CP/CPPS.

Brain area (abbreviations)	Brain area (full name)	Number of studies	Functional/structure	Sign	Literature name
ACC.L	Anterior cingulate cortex	Four studies	Functional/structure	↓	S2, S4, S5, S6
ACC.R	Anterior cingulate cortex	Three studies	Functional/structure	↓	S2, S4, S6
INS.R	Insula	Three studies	Functional	↓	S1, S3, S4
PoCG.L	Postcentral gyrus	Three studies	Functional	—	S1, S7, S9
PreCG.L	Precental gyrus	Three studies	Functional	—	S1, S7, S9
PCUN.L	Precuneus	Three studies	Functional	—	S1, S7, S9
INS.L	Insula	Two studies	Functional	↓	S1, S4
DLPFC.R	Dorsolateral prefrontal cortex	Two studies	Functional/structure	↑	S1, S5
THA.R	Thalamus	Two studies	Functional	↑	S4, S6
mPFC.L	Medial prefrontal cortex	Two studies	Functional/structure	↑	S5, S8
ORBmid.R	Middle frontal gyrus, orbital part	Two studies	Functional	↓	S7, S9
DCG.L	Median cingulate and paracingulate gyri	Two studies	Functional	↑	S7, S9
DCG.R	Median cingulate and paracingulate gyri	Two studies	Functional	↑	S7, S9
SMA.L	Supplementary motor area	Two studies	Functional	—	S7, S9
SMA.R	Supplementary motor area	Two studies	Functional	—	S7, S9
PCG.L	Posterior cingulate gyrus	Two studies	Functional	—	S7, S9
SPG.L	Superior parietal gyrus	Two studies	Functional	—	S7, S9
SMG.R	Supramarginal gyrus	Two studies	Functional		S7, S9
PCL.L	Paracentral lobule	Two studies	Functional	↑	S7, S9
STG.R	Superior temporal gyrus	Two studies	Functional		S7, S9
MTG.L	Middle temporal gyrus	One study	Functional/structure	—	S1
MTG.R	Middle temporal gyrus	One study	Functional/structure	—	S1
Frontal. L	Orbital frontal cortex	One study	Functional/structure	—	S1
Frontal.R	Orbital frontal cortex	One study	Functional/structure	—	S1
MFG.L	Middle frontal gyrus	One study	Structure		S2
VPC.R	Ventrolateral parietal cortex	One study	Functional/structure	—	S1
VPC.L	Ventrolateral parietal cortex	One study	Functional/structure	—	S1
PPC.L	Posterior parietal cortex	One study	Functional/structure	—	S1
PPC.R	Posterior parietal cortex	One study	Functional/structure	—	S1
IFGoperc.R	Inferior frontal gyrus, opercular part	One study	Functional	—	S3
THA.L	Thalamus	One study	Functional/structure	—	S1
Brainstem. R	Brainstem	One study	Functional		S4
Brainstem. L	Brainstem	One study	Functional	—	S4
mPFC.R	Medial prefrontal cortex	One study	Functional		S4
HIP.R	Hippocampus	One study	Functional/structure	—	S5
Cerebellum anterior lobe.L	Anterior cerebellum lobe.	One study	Functional	—	S8
PoCG.R	Postcentral gyrus	One study	Functional/structure	—	S1
PreCG.R	Precental gyrus	One study	Functional/structure	—	S1
PHG.R	Parahippocampal gyrus	One study	Functional	—	S9

L: left hemisphere; R: right hemisphere.

**Table 4:** ALE analysis results.

Cluster #	MNI coordinates X Y Z			ALE (×10^−3^)	*p*	Z	Hemisphere	Label
1	48	2	−6	9.10	6.90*E*−05	3.811792	R	Insula
	52	2	−2	9.07	7.11*E*−05	3.804371	R	Superior temporal gyrus
	56	14	8	9.06	9.36*E*−05	3.735753	R	Precentral gyrus
	42	−4	4	9.06	9.36*E*−05	3.735753	R	Claustrum
	50	−6	2	9.06	9.36*E*−05	3.735753	R	Insula
	66	−2	6	9.06	9.36*E*−05	3.735753	R	Superior temporal gyrus
	34	24	0	8.71	1.14*E*−04	3.68467	R	Insula
	44	14	−4	8.33	1.99*E*−04	3.541374	R	Insula

2	8	14	30	16.28	1.09*E*−07	5.182729	R	Cingulate gyrus
	−6	28	24	14.10	1.52*E*−06	4.667913	L	Cingulate gyrus
	−10	18	24	9.53	3.96*E*−05	3.947112	L	Cingulate gyrus

L: left hemisphere; R: right hemisphere.

## Data Availability

The data used in this study are all included in this paper and are open to all readers.

## Supplementary Materials

Supplementary Materials Supplementary Table 1: Chinese search terms.
