# Supplementary material for: Neuroimaging Studies of Chronic Prostatitis/Chronic Pelvic Pain Syndrome
Source: Pain Res Manag. 2022 May 4;2022:9448620. doi: 10.1155/2022/9448620 (PMC9095382; doi:10.1155/2022/9448620)
Supplement: Supplementary Materials — Supplementary Table 1: Chinese search terms. [file 9448620.f1.zip › 9448620.f1/Supplementary Materials.docx]

Supplementary Table 1: Chinese search terms

| English search terms | Matched Chinese search terms |
| --- | --- |
| Chronic prostatitis | 慢性前列腺炎 |
| Chronic pelvic pain syndrome | 慢性盆腔疼痛综合征 |
| Chronic nonbacterial prostatitis | 慢性非细菌性前列腺炎 |
| Prostatodynia | 前列腺痛 |
| Magnetic resonance imaging | 核磁共振 |
| Brain | 脑 |
